# Indication-based clinical decision support for inpatient urine testing: impacts on utilization, appropriateness, and antibiotic prescribing

**DOI:** 10.1017/ash.2026.10805

**Published:** 2026-07-24

**Authors:** Mario Luis Valdez Imbert, Allyson Larcena Tipgos, Majd Alsoubani, Maureen Campion, Rachel Erdil, Shira Doron, Gabriela Andujar Vazquez, Kap Sum Foong

**Affiliations:** 1 Infectious Diseases, Tufts Medical Center, Boston, USA; 2 Infectious Diseases, Dartmouth-Hitchcock Clinic: Dartmouth Hitchcock Medical Center, Lebanon, USA

## Abstract

Across a healthcare system, an indication-based clinical decision support (CDS) tool for inpatient urine testing immediately reduced urinalysis-with-reflex-to-culture (−62.7/1,000 patient-days) and urine culture ordering, while increasing urinalysis utilization(41.4/1,000 patient-days). Intervention effect was not fully sustained. Ordering appropriateness remained low, and antibiotics were often prescribed without documented symptoms, suggesting CDS alone is insufficient.

## Introduction

Inappropriate urine testing in hospitalized patients contributes to unnecessary treatment of asymptomatic bacteriuria, as cultures obtained without urinary symptoms detect colonization rather than infection, driving avoidable antimicrobial exposure.^1,2^ Despite national guideline recommendations against screening for and treatment of asymptomatic bacteriuria in most populations, unnecessary urine testing remains common across acute care settings.^1^ Reducing unnecessary urine testing is therefore an important target for diagnostic stewardship.^2^

Diagnostic stewardship aims to optimize test ordering, processing, and interpretation to improve clinical care and antimicrobial use.^2^ Clinical decision support (CDS) integrated into computerized physician order entry(CPOE) has shown promise in reducing urine culture utilization.^3^ However, the impact of indication-based CDS on overall urine testing patterns and on documentation-supported appropriateness of test ordering and antibiotic prescribing remain unclear.

In August 2024, Tufts Medicine implemented an indication-based CDS tool for inpatient urine testing. We evaluated its impact on utilization and assessed postimplementation ordering appropriateness and antibiotic prescribing through targeted chart review.

## Methods

### Setting

We conducted a quasi-experimental interrupted time series (ITS) study across the Tufts Medicine healthcare system, a 1,039-bed system comprising one tertiary-care academic hospital and two community hospitals. The study period spanned August 2023 through July 2025, including 12 months before and after CDS implementation in August 2024. No other concurrent stewardship interventions or electronic health record changes affecting urine testing occurred during the study period.

### Intervention

**Figure 1.**
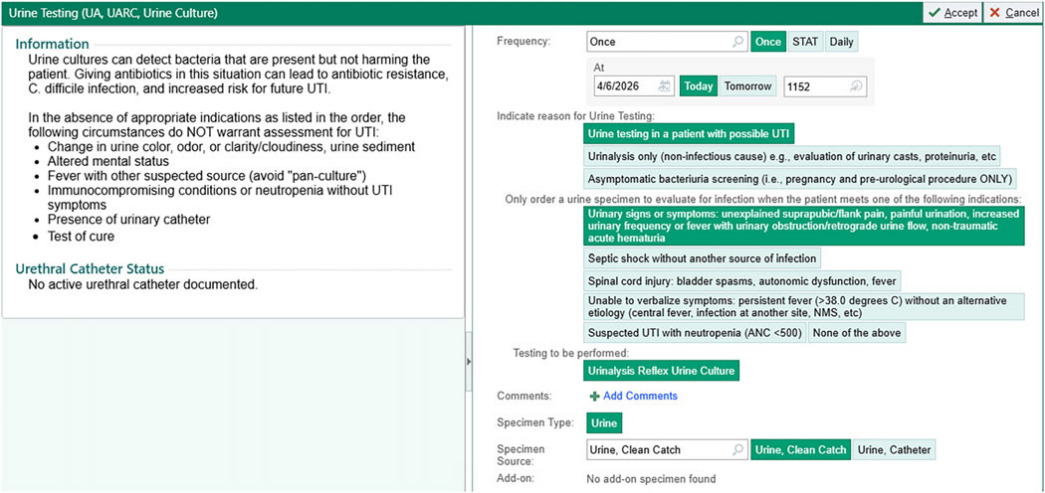
Figure 1 long description. Clinical decision support tool interface and indication selection for inpatient urine testing.

The CDS tool was integrated into the CPOE workflow for inpatient urinalysis alone (UA), urinalysis-with-reflex-to-culture (UARC), and urine culture alone (UCx), requiring providers to select a clinical indication prior to order completion. The CDS presented educational messaging on inappropriate indications for urine culturing but did not trigger indication-specific advice or warnings. For orders not meeting criteria for UARC or UCx, UA was available as an alternative. Indications were derived from institutional guidance aligned with national recommendations (Figure 1). Prior to implementation, clinicians were notified through a systemwide email that included screenshots of the CDS orders and ordering guidance, with targeted emails to department chairs to facilitate distribution.

### Data sources and outcomes

Urine testing data were extracted from the electronic health record and aggregated monthly. Patient-day denominators were obtained from hospital census data. Primary outcome was the monthly utilization rate per 1,000 patient-days for UA, UARC, and UCx. Secondary outcomes included trend(slope) changes in utilization following CDS implementation.

### Statistical analysis

Segmented regression was used to perform ITS analysis, estimating baseline trends, immediate level changes at implementation, and postintervention slope changes. Level changes are reported as tests per 1,000 patient-days and slope changes as the change per 1,000 patient-days per month, each with 95% confidence intervals(CI). Monthly testing rates were modeled assuming a normal distribution with an identity link. Statistical significance was defined as *P* < .05. Analyses were performed using SPSS version 25 (IBM, Armonk, NY, USA).

### Chart review

We performed a patient-level chart review at Tufts Medical Center to assess ordering appropriateness and downstream antibiotic prescribing. We focused on UARC because it is the most frequently ordered and most commonly misordered urine test at our institution. We reviewed a random sample of 153 unique adult inpatients with UARC orders placed during April 2025. Repeat orders from the same patient were excluded. Two independent infectious-disease-trained reviewers (A.L.T., M.V.I.) applied predefined appropriateness criteria, piloted on a subset of cases prior to formal review, and assessed selected CDS indications, documentation of urinary symptoms or qualifying criteria, clinical justification for testing, and whether antibiotics were initiated for presumed urinary tract infection (UTI). Discrepancies were adjudicated by a third investigator (M.A.). This study was approved by the Institutional Review Board of Tufts Medical Center.

## Results

During the study period, 124,762 urine tests were ordered across 225,614 patient-days. There were no significant preintervention trends for UA, UARC, or UCx.

**Figure 2.**
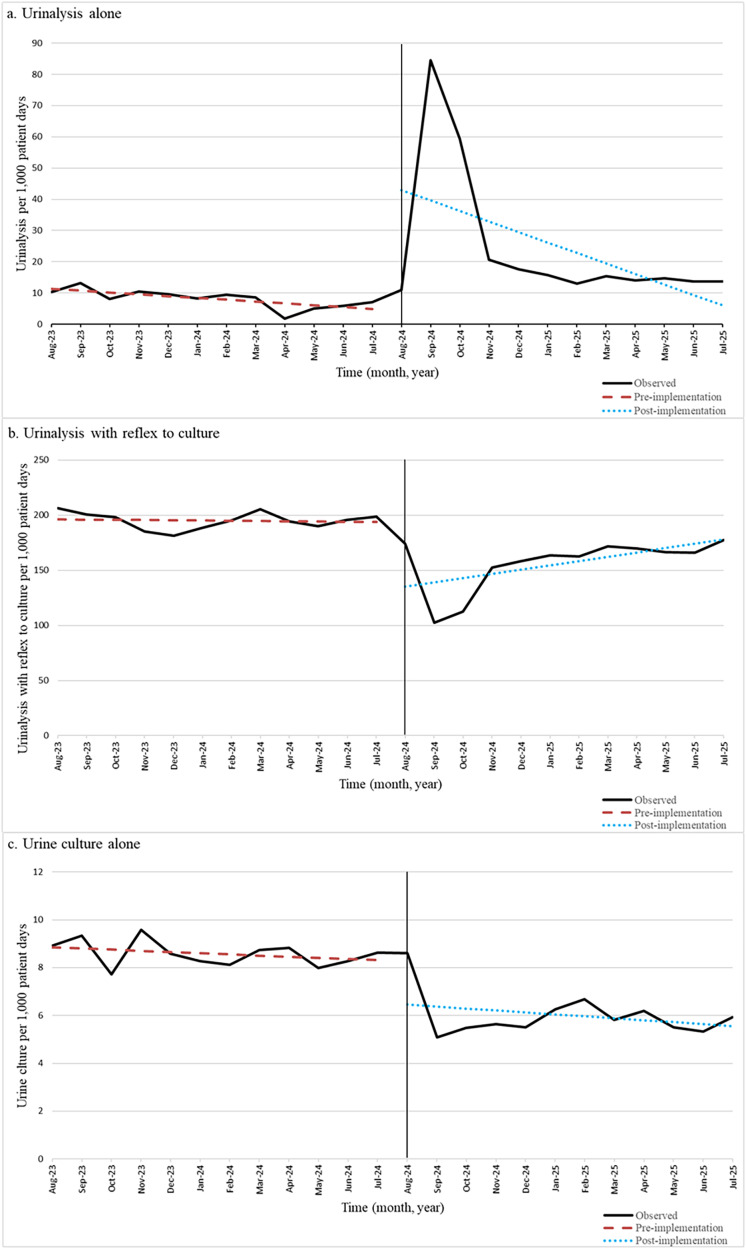
Monthly urine testing rates before and after implementation of clinical decision support tool at Tufts Medicine, August 2023–July 2025.

ITS analysis demonstrated an immediate increase in UA utilization after CDS implementation (41.4; 95% CI, 20.1 to 62.8), with no significant postintervention slope change (−2.8; 95% CI, −5.9 to 0.3). In contrast, UARC utilization showed an immediate decrease (−62.7; 95% CI, -−85.4 to −39.9), followed by a modest increase in postintervention slope (4.1; 95% CI, 0.8 to 7.4). UCx utilization also demonstrated an immediate decrease (−1.8; 95% CI, −2.9 to −0.7), with no significant postintervention slope change(−0.03; 95% CI, −0.2 to 0.1) (Figure 2; Supplemental Table 1).

Of the 153 reviewed UARC orders, 46.4% originated in the emergency department (ED), 41.2% in intensive care units, and 12.4% on inpatient floor units. Documentation supported appropriate testing in 9.9% of ED encounters, 19.2% of inpatient floor encounters, and 27.8% of intensive care unit encounters. Antibiotics for presumed UTI were initiated in 29 patients (19%), whereas only 9 (5.9%) had documentation consistent with symptomatic infection.

## Discussion

Implementation of an indication-based CDS tool for inpatient urine testing was associated with immediate, intended reductions in UARC and UCx ordering. However, these effects were incomplete and not fully sustained. UARC utilization partially rebounded, and UA ordering increased immediately after implementation, suggesting substitution toward less restricted testing rather than a true reduction in low-value testing. Indication-based CDS alone was therefore insufficient to produce sustained, guideline-concordant practices in our setting.

Several factors may explain these results. First, our intervention functioned as a required prompt without a hard stop, and softer interventions are more vulnerable to bypass or workaround behavior,^4,5^ potentially explaining the immediate reduction in culture-based testing without consistent improvement in appropriateness. Second, the rise in UA ordering suggests substitution to a less restricted test. For clinicians remain concerned about missing infection, indication-based prompts may shift ordering without changing the underlying diagnostic threshold, consistent with qualitative work identifying diagnostic uncertainty, fear of undertreatment, and ingrained practice norms as barriers to stewardship.^6^ Third, required CDS fields may also fail to capture clinical reasoning when the tool is perceived as administrative rather than a meaningful decision aid, as alert fatigue promotes routinized selection.^7,8^ In this context, requiring indication selection does not ensure accurate indication selection.

Our audit findings reinforce this. Documentation-supported appropriateness remained low across inpatient settings, particularly in the ED, where prior work has identified ED ordering as a strong predictor of inappropriate urine culturing.^9^ Antibiotics for presumed UTI were often initiated without documented symptoms. These findings suggest that interventions targeting test ordering alone may have limited downstream effect unless paired with complementary strategies aimed at interpretation and prescribing, such as prospective audit and feedback.^2,10^

This study has several limitations. It was a single healthcare system study, and chart review was limited to a sample of 153 postimplementation UARC orders at Tufts Medical Center, which may limit generalizability. Although our ITS design uses each period as its own control, accounting for stable or gradually changing population characteristics, we did not tabulate baseline characteristics across periods, and interrater agreement for the chart review was not formally assessed. Appropriateness assessments were based on chart documentation and may have unrecorded clinical reasoning. Lastly, because review was performed only after implementation, we could not directly compare pre and postintervention appropriateness.

In conclusion, implementation of an indication-based CDS tool produced immediate reductions in culture-based urine testing, offset by test substitution, limited sustainability, low documentation-supported appropriateness, and antibiotic treatment without documented symptoms. These findings suggest that indication-based CDS, particularly without a hard stop or complementary stewardship measures, may be insufficient to achieve sustained improvement in urine testing and downstream prescribing. Multifaceted approaches combining diagnostic stewardship with behavioral, workflow, and prescribing-focused interventions are likely needed.

## Supporting information

10.1017/ash.2026.10805.sm001 Valdez Imbert et al. supplementary material Valdez Imbert et al. supplementary material

## Data Availability

Data is available upon request to the corresponding author.

## Supplementary material

The supplementary material for this article can be found at https://doi.org/10.1017/ash.2026.10805.

## Previous presentation

This work was presented at SHEA Spring in April 2026 in Chicago, Illinois.

## Figure 1. Long description

A table detailing urine testing guidelines and criteria for hospitalized patients. The table has multiple sections including Information, Urethral Catheter Status, Frequency, Indicate reason for Urine Testing, Only order a urine specimen to evaluate for infection when the patient meets one of the following indications, Testing to be performed, Comments, Specimen Type, Specimen Source, and Add-on. The Information section warns about the detection of non-harmful bacteria and the risks of unnecessary antibiotic use. It lists circumstances where urine testing is not warranted. The Urethral Catheter Status indicates no active urethral catheter. The Frequency section allows selection of testing frequency and date. The Indicate reason for Urine Testing section lists various reasons such as Urine testing in a patient with possible UTI, Urinalysis only, Asymptomatic bacteriuria screening, and others. The Only order a urine specimen section lists indications for urine testing including urinary signs or symptoms, septic shock, spinal cord injury, inability to verbalize symptoms, and suspected UTI with neutropenia. The Testing to be performed section specifies Urinalysis Reflex Urine Culture. The Comments section allows adding comments. The Specimen Type and Source sections specify the type and source of the urine specimen. The Add-on section indicates no add-on specimen found.

Navigate back to Figure 1..
