# Supplementary material for: Indication-based clinical decision support for inpatient urine testing: impacts on utilization, appropriateness, and antibiotic prescribing
Source: Antimicrob Steward Healthc Epidemiol. 2026 Jul 24;6(1):e229. doi: 10.1017/ash.2026.10805 (PMC13419427; doi:10.1017/ash.2026.10805)
Supplement: Valdez Imbert et al. supplementary material [file S2732494X26108055sup001.docx]

**Supplemental Table 1.** Interrupted time series estimates of level and trend changes in monthly urine testing utilization after CDS implementation

| Urine test |  | Parameter estimate (95% CI) | *p* value |
| --- | --- | --- | --- |
| UA | Intercept pre-intervention | 11.9 (-4.1 to 27.9) | 0.144 |
|  | Pre-intervention segment slope | -0.6 (-2.8 to 1.6) | 0.599 |
|  | Level change post-intervention | 41.4 (20.1 to 62.8) | <.001 |
|  | Change in slope post-intervention | -2.8 (-5.9 to 0.3) | 0.076 |
| UARC | Intercept pre-intervention | 196.5 (179.4 to 213.5) | <.001 |
|  | Pre-intervention segment slope | -0.2 (-2.5 to 2.1) | 0.853 |
|  | Level change post-intervention | -62.7 (-85.4 to -39.9) | <.001 |
|  | Change in slope post-intervention | 4.1 (0.8 to 7.4) | 0.014 |
| UCx | Intercept pre-intervention | 8.9 (8.1 to 9.7) | <.001 |
|  | Pre-intervention segment slope | -0.1 (-0.2 to 0.1) | 0.395 |
|  | Level change post-intervention | -1.8 (-2.9 to -0.7) | 0.002 |
|  | Change in slope post-intervention | -0.03 (-0.2 to 0.1) | 0.699 |

Abbreviation: CDS, clinical decision support; CI, confidence interval; UA, urinalysis alone; UARC, urinalysis-with-reflex-to-culture; UCx, urine culture alone.
